# Role of PKC and ERK Signaling in Epidermal Blistering and Desmosome Regulation in Pemphigus

**DOI:** 10.3389/fimmu.2019.02883

**Published:** 2019-12-06

**Authors:** Desalegn Tadesse Egu, Daniela Kugelmann, Jens Waschke

**Affiliations:** Chair of Vegetative Anatomy, Faculty of Medicine, Institute of Anatomy, LMU Munich, Munich, Germany

**Keywords:** PKC signaling, ERK signaling, desmosomes ultrastructure, pemphigus, electron microscope analysis

## Abstract

Desmosomes reinforce cohesion of epithelial cells at the interface between adjacent cells. They include the cadherin-type adhesion molecules desmoglein 1 (Dsg1) and Dsg3. Pemphigus vulgaris (PV) is an autoimmune disease in which circulating autoantibodies (PV-IgG) targeting Dsg1 and 3 cause characteristic epidermal blister formation. It has been shown that PV-IgG binding induced activation of kinases such as ERK and PKC, and inhibition of these signaling pathways prevented loss of cell cohesion in cell cultures. However, the role of Erk and PKC in blister formation and regulation of desmosome ultrastructure in human skin are unknown. Accordingly, we assessed the role of PKC and ERK signaling pathways in blister formation and regulation of desmosome ultrastructure in human epidermis. Here we performed electron microscopy analyses using human skin explants injected with PV-IgG together with inhibitors for PKC or ERK signaling. Inhibition of PKC was not effective to prevent suprabasal blister formation or ultrastructural alterations of desmosomes. In contrast, inhibition of ERK signaling significantly ameliorated blister formation and decrease in the number of desmosomes whereas shortening and splitting of desmosomes and keratin filament insertion were not different from samples treated with PV-IgG alone. However, apical desmosomes between basal and suprabasal cells remained unaltered when ERK signaling was inhibited. Therefore, our results show that inhibition of ERK but not PKC signaling appears to be effective to ameliorate blistering and alterations of desmosome ultrastructure triggered by PV-IgG in human skin.

## Introduction

Desmosomes reinforce cohesion of epithelial cells at the interface between adjacent cells (1). They include different isoforms of the cadherin-type adhesion molecules such as desmoglein 1 (Dsg1) and Dsg3 (2). Pemphigus vulgaris (PV) is an autoimmune disease in which circulating autoantibodies (PV-IgG) targeting Dsg1 and/or 3 cause skin blistering and erosions of the epithelial lining of the oral cavity (3, 4).

The pathomechanisms underlying loss of cell contacts of keratinocytes have been widely accepted to be caused by steric hindrance and signaling (5). Direct inhibition of desmoglein interaction is caused by the interference of the autoantibodies through binding to the extracellular adhesive domains of desmogleins (6) which involves disruption of interaction of Dsg3 resulting in loss of cellular cohesion (7).

On the other hand, a large body of experimental data has confirmed that pharmacologic inhibition of signaling pathways ameliorated intraepidermal cleft formation *in vivo* and cell dissociation *in vitro* (8–10). Signaling cascades may be activated downstream of antibody binding and as a result, it is believed that both mechanisms may synergistically orchestrate blister formation (4, 11, 12).

Accordingly, several cellular responses to autoantibody binding in pemphigus have been attributed to different signaling pathways among which are mitogen-activated protein kinases (MAPKs) such as p38MAPK and extracellular signal-regulated kinases (ERK1/2), as well as Rho GTPase, epidermal growth factor receptor (EGFR), Rous sarcoma-related kinase (Src) and protein kinase C (PKC) (12, 13). However, no precise signaling cascade has been defined to date to depict how this mechanism exactly operates.

p38MAPK signaling is one of the most extensively studied signaling pathways in pemphigus pathology. It is activated secondary to PV-IgG binding and its phosphorylation is detected in lesioned skin of PV patients (14) and in keratinocyte cell cultures treated with PV-IgG (15, 16). Interestingly, pharmacologic inhibition of p38MAPK effectively prevented all PV-IgG-induced features of the disease in keratinocyte cell cultures (15, 17), animal models (8), and in *ex vivo* human skin (18) but not in an *ex vivo* model of human mucosa (19). As a result, p38MAPK is believed to be central in PV pathogenesis, at least with respect to skin blister formation.

Besides, EGFR and ERK1/2 have been implicated in loss of keratinocyte cohesion based on studies in cultured cells (17, 20, 21). Dsg1-dependent suppression of EGFR/ERK signaling pathway has been shown to promote epidermal differentiation linking this signaling pathway to pemphigus autoantigens (22). Similarly, activation of ERK downstream of EGFR was shown to trigger signaling cascades that lead to actin reorganization and as a result matrix degradation (23) in the absence of Dsg1 (24). In line with this, it was shown recently that EGFR was activated following PV-IgG incubation in Src- and EGFR-kinase-dependent manner but independent of Dsg1 whereas ERK1/2 was activated only when antibodies against Dsg1 were present (17, 21). Moreover, Suppression of Tumorigenicity 18 (ST18) may also enhance the susceptibility of keratinocytes to PV-IgG-induced loss of cell adhesion via up-regulation of ERK suggesting that ERK may play a role in the regulation of desmosomes in pemphigus (25).

PKC is a downstream target of Phospholipase C (PLC), an isoenzyme which through its downstream substrates causes an increase in intracellular Ca^2+^ concentration, and activates PKC (26). In an earlier study, it has been proposed that intracellular signaling events secondary to PV-IgG induced PLC activation may be mediated by PKC (27). Another study also demonstrated that desmosomes modulated their adhesive states as a direct effect of PKC signaling (28). Furthermore, inhibition of PKC was shown to be sufficient to mitigate the PV-IgG triggered dys-cohesive effects through maintaining a hyper-adhesive state of desmosomes in keratinocyte cell culture (29). This is supported by findings showing that PKC is involved in PV-IgG-induced Dsg3 depletion in human skin and contributes to blister formation in a passive mouse transfer model *in vivo* (30, 31).

Taken together, several laboratories have generated ample data on the PV-IgG-mediated roles of PKC and ERK signaling in cultured cells and animal models. Despite the fact that several signaling mechanisms are activated following incubation with pemphigus autoantibodies, inhibition of a single signaling pathway is often protective in neonatal mice and cultured keratinocytes. Therefore, these models do not allow to integrate the different pathways and to clarify their relative contribution to pemphigus pathogenesis.

Unfortunately, no data is available to date on the effects of these signaling molecules on blister formation and desmosome ultrastructure in human skin samples. In this study, therefore, we employed an *ex vivo* human skin culture model to evaluate the effect of these signaling pathways on epidermal acantholysis after PV-IgG treatment.

## Materials and Methods

### Tissue Culture

Skin biopsy specimens were harvested from body donors without a history of skin lesions among those received by the Institute of Anatomy and Cell Biology, Ludwig-Maximilian-Universität München, Germany. Written informed consent was obtained from body donors for use of tissue samples in research. Body donors which arrived within 24 h after decease were considered for the study. Each skin piece, ~4 × 4 cm size, was excised from the shoulder region, stripped of fat and excess connective tissue, and was divided into 2 × 2 cm pieces for injection of pemphigus autoantibodies with or without the inhibitors of PKC or ERK signaling used in the study.

An intradermal injection was performed using a 30G syringe, and 50 μl of PV-IgG purified from a PV patient serum was injected into the sample. The PV-IgG contained antibodies against Dsg3 (ELISA score: 174.64 U/ml) and Dsg1 (ELISA score: 168.1 U/ml). Controls were injected with IgG from a healthy volunteer. For PKC or ERK1/2 inhibition assay, samples were treated with 50 μl of 1 μM bisindolylmaleimide X hydrochloride (Bim-X) (Enzo Life Sciences, Inc., NY, USA) or 5 μM UO126 (New England Biolabs, Ipswich, USA), respectively, 1 h prior to PV-IgG injection. All other samples were treated with DMSO (dimethyl sulphoxide) in PBS as vehicle control. Samples were incubated floating in Dulbecco's modified Eagle's medium (DMEM) with the epidermis facing upwards at liquid- air interface without additional support. The samples were cultured in a humidified atmosphere of 95% air and 5% CO_2_ at 37°C for 24 h. Viability assay was done using MTT assay [1-(4,5- dimethylthiazol-2-yl)-3,5 diphenylformazan] for all conditions before and after incubation. A piece from each sample was heat-inactivated at 65°C for 30 min as a negative control. Each condition was evaluated in biopsies from at least three different body donors. Shear stress was gently applied with a rubber head in the same manner for all conditions. Finally, specimens were cut into two asymmetric parts and processed either for hematoxylin and eosin (HE) and immunostaining, or electron microscopy analyses.

### Histology

For histological analysis, samples were embedded in tissue freezing medium (Leica Biosystems, Nussloch, Germany) and 7 μm thick serial sections were made using a cryostat microtome (HM 500 OM MICROM International GmbH, Walldorf, Germany) until the entire sample was sliced. The resulting sections were stained with HE according to standard protocols and mounted using DPX Mountant for histology (Sigma-Aldrich, St. Louis, USA). For morphometric analysis, every tenth section was considered. Images were captured at 200× magnifications using a light DMI8-Microscope (Leica, Mannheim, Germany).

### Immunofluorescence

Randomly drawn slices were used for immunostaining. Sections were heated at 60°C (30 min), fixed with 2% paraformaldehyde in PBS (20 min), permeabilized with 0.1% Triton X-100 (45 min), and blocked with 3% bovine serum albumin (BSA) and 1% normal goat serum (1 h). Antibodies used as primary antibodies were: mouse anti-Dsg1mAb (p124, 1:10 dilution, Progen, Heidelberg, Germany), mouse anti-Dsg3 mAb (5G11, 1:100 dilution, ThermoFisher Scientific Carlsbad, CA, USA). All primary antibodies were incubated overnight at 4°C. Cy3-conjugated goat-anti-mouse secondary antibody (Dianova, Hamburg, Germany) was applied for 1 h at room temperature protected from light. Some slides were stained with goat-anti-mouse secondary antibody alone as a negative control (data not shown). Then, DAPI (4′, 6-diamidino-2-phenylindole) was applied for 10 min to help visualize the nuclei.

In addition, some tissue slices were stained with goat-anti-human secondary antibody to verify that autoantibodies were deposited in the epidermis in the absence and presence of inhibitors (data not shown). Finally, slides were mounted with 1.5% n-propyl gallate in glycerol and images were captured using a SP5 confocal microscope with an X63 NA 1.4 PL APO objective (Leica). Brightfield mode was used to identify the area of the epidermis.

### Electron Microscopy

Portions of the injected skin samples were further sliced into small pieces of ~2 mm size. These were fixed in 2.5% glutaraldehyde in PBS and then washed in PBS. They were post-fixed with 2% osmium tetroxide and dehydrated through an ascending ethanol series. The samples were subsequently cleared in propylene oxide and were infilitrated with EPON 812 resin (SERVA Electrophoresis GmBH, Heidelberg, Germany), and cured at 40°C for 20 h and then at 60°C for another 24 h. The resulting blocks were trimmed and initially semithin sections of 0.99 μm thickness were stained with Toluidine blue for light microscopy to examine the area to be considered for ultrathin sectioning, and ultimately 60 nm thick unltrathin sections were made, both with an ultramicrotome (Reichert-Jung Ultracut E, Optische Werke AG, Vienna, Austria) using a diamond knife (*DiATOME* Electron Microscopy Sciences, Hatfield, PA). Silver appearing sections were harvested and mounted on 150 mesh copper/rhodium grids (Plano GmbH, Wetzlar, Germany) and were then contrasted using alcoholic uranyl acetate and lead citrate. Images were acquired with a Libra 120 transmission electron microscopy (Carl Zeiss NTS GmbH, Germany) equipped with a SSCCD camera system (TRS, Olympus, Tokyo, Japan).

### Quantification of PV-IgG Autoantibody Effects

HE stained serial sections were evaluated for blistering as well as cleft formation. The presence and absence as well as the size of blister was assessed in a method previously described (18). The occurrence of blister was scored in whole numbers ranging from 0 to 4 in accordance with the following criteria: 0 (no blister), cleft covering 1–25, 26–50, 51–75, 76–100% of section length as 1, 2, 3, or 4, respectively. In addition, cleft length was evaluated using a straight hand tool from *ImageJ* software (Wayne Rasband; *https://imagej.nih.gov/ij/**)*. This was done by drawing a line along the area of the epidermis between detached basal and suprabasal cells until the two ends came in contact with intact cells. Clefts formed by detachment of cells as few as two were also included in the measurement. The resulting number of pixels was expressed as percentage cleft length which is the ratio of the total cleft length to the entire length of the epidermis in a given tissue slice.

Moreover, the degree of fragmentation of Dsg1 as well as Dsg3 staining was evaluated similarly to the criteria described previously (30). Accordingly, the mean fluorescence intensity of the basal cells of the epidermis was divided by that of the upper most layers for each condition and the result was expressed as the ratio of the basal fluorescence intensity (Fb) to that of the superficial layers (s), thus Fb/s.

For electron microscope analysis, micrographs taken from basal and suprabasal layers of the epidermis were analyzed at 4,000 × magnification. Then, 16–25 micrographs were randomly selected from each body donor, and for each condition ~250 desmosomes were evaluated using *ImageJ*. The number of desmosomes was counted as previously described (18) and the result was expressed as the number of desmosomes per μm membrane length. This helps to account for the variability in the number of cells present in each micrograph. The size of desmosomes was measured as the distance between the two ends of a desmosome and expressed in μm.

### Statistical Analysis

A mean value per donor was considered for statistics and data are presented as mean ± the standard error of the mean (SEM), the latter represented by error bars. Comparison of data was done using one-way analysis of variance with subsequent Bonferroni *post-hoc* test (for Gaussian-distributed samples). The analysis was performed using Prism (GraphPad Software V6.02, La Jolla, CA, USA). A value of *P* < 0.05 was assumed to indicate statistical significance.

## Results

**Figure 1:**
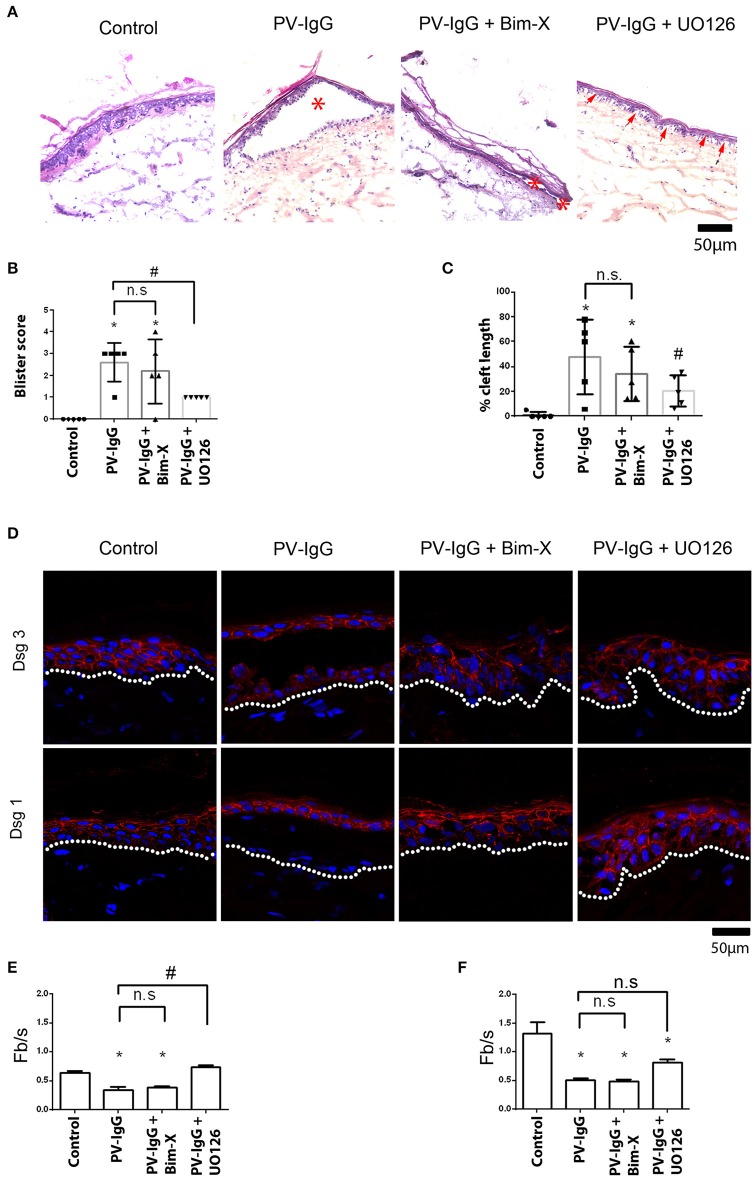
Histological analysis showing suprabasal cleft formation and Dsg3 depletion in skin explant culture. **(A)** HE staining, *shows blisters, whereas arrows show small cleft formation between basal cells. **(B)** Blister score represents the size of blisters formed, and **(C)** represents cleft length as percentage of the length of section. **(D)** Immunostaining with Dsg1 and Dsg3 antibodies. Dsg1 and 3 show a fragmented distribution in PV-IgG-treated cells and complete depletion of Dsg1 in blister floor. When UO126 was used in addition, Dsg1 staining was partially preserved. **(E)** Quantification of effects on Dsg1 localization. **(F)** Quantification of effects on Dsg3 localization. *n* = 3–5 for each condition Scale bar = 50 μm. *P < 0.05 vs. control, ^#^*P* < 0.05 vs. PV-IgG.

Human skin biopsies harvested from body donors in <24 h after decease received transdermal injections of a PV-IgG autoantibody fractions with or without inhibitors of either PKC or ERK signaling. After 24 h incubation period, shear force was applied to all samples and processed for histology as described above. Presence or absence as well as the size of the resulting blisters were then evaluated (Figures 1A,B). Accordingly, no blister formation was observed in control samples, whereas samples treated with PV-IgG only significantly developed large blisters (asterisks in Figure 1A, score: 2.6 ± 0.4). However, in samples treated with PV-IgG in combination with UO126 to inhibit MEK signaling, suprabasal blistering was significantly reduced (score: 1.0 ± 0.0). On the other hand, samples incubated with PV-IgG in the presence of Bim-X to inhibit PKC displayed blisters with a score not significantly different from samples treated with PV-IgG alone (asterisks in Figure 1A, 2.2 ± 0.66). Interestingly, in samples pretreated with UO126 followed by PV-IgG, small clefts were visible in the lateral interfaces of adjacent basal cells (arrows in Figure 1A), although suprabasal blisters did not occur. We then evaluated whether these microblisters observed differ significantly from other samples used in the study. To this end we measured cleft length for each condition and added values for each sample (Figure 1C). As a result no cleft formation was evident in control samples but all other samples showed epidermal clefts to a varying extent. PV-IgG-treated samples as well as those treated with both PV-IgG and Bim-X displayed large clefts (47.82 ± 10.97% of length of section and 34.11 ± 7.97% of length of section, respectively). However, samples which received PV-IgG in combination with UO126 showed clefts with significantly reduced length (20.76 ± 4.60% of length of section). In addition, immunostaining showed a fragmented distribution of Dsg1 and Dsg3 in samples treated with PV-IgG, when compared to controls (Figure 1D). In addition, Dsg1 was completely depleted in the blister floor after incubation with PV-IgG. In contrast, in presence of UO126 but not Bim-X, staining of Dsg1 and Dsg3 was less fragmented, and especially between basal and suprabasal cells Dsg1 staining was preserved. Quantification of immunostaining signals demonstrated that UO126 but not Bim-X abolished loss of Dsg1 staining intensity whereas changes for Dsg3 were not significant for both inhibitors compared to samples incubated with PV-IgG alone (Figures 1E,F).

**Figure 2:**
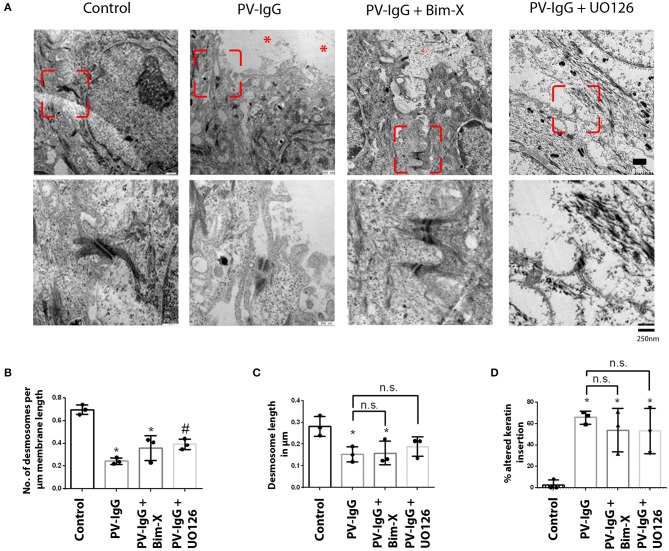
Ultrastructural alterations in desmosomes after PV-IgG injection as revealed by transmission electron microscopy. **(A)** Electron-micrograph showing reduction in desmosome number and length as well as reduced keratin association with desmosomal plaque after PV-IgG treatment (*shows blister areas). Decrease in desmosome number is significantly rescued only in samples pretreated with UO126. Scale bars for upper and lower panels, 500 and 250 nm, respectively. **(B)** Desmosome number quantified and expressed as number per μm membrane length, **(C)** desmosome size expressed as length of desmosomes in μm. **(D)** Ratio of keratin filaments uncoupled from desmosomes expressed as percentage of altered keratin insertion. *n* = 3 for each condition. *P < 0. 05 vs. control, ^#^*P* < 0.05 PV-IgG, respectively.

**Figure 3:**
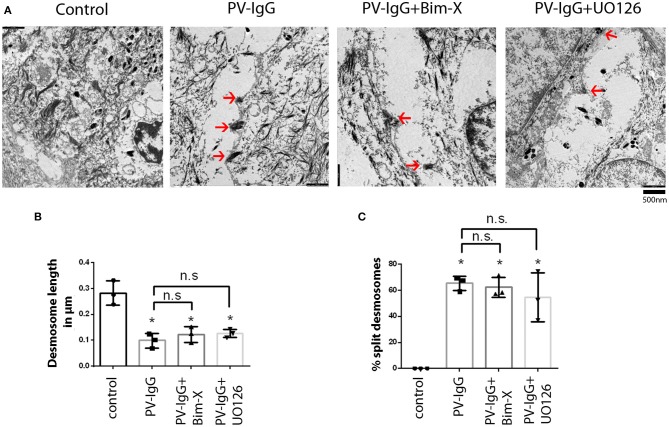
Split desmosomes after incubation with PV-IgG. **(A)** Electron-micrograph of split desmosomes with reduced plaques as well as reduced keratin associations. Arrows indicate split desmosomes. Scale bar: 500 nm. **(B)** Length of split desmosomes as compared to normal controls. **(C)** Ratio of split desmosomes expressed as percentage of total desmosomes in the vicinity of blister. *n* = 3. *P < 0. 05 vs. control.

Ultrastructural analysis of desmosomes in skin subjected to PV-IgG has shown that desmosomes are reduced in number and size, were split and dissociated from keratin filaments, all of which was modulated by inhibition of p38 MAPK signaling (18). Hence, we performed similar analyses to check whether these ultrastructural hallmarks of desmosomes, which similarly were described in patients' lesions (32), were abrogated by inhibition of PKC or ERK signaling (Figures 2, 3). Compared to controls (0.69 ± 0.02/μm), the number of desmosomes (Figure 2B) was significantly reduced in samples treated with PV-IgG alone as well as in those samples which received the PKC inhibitor Bim-X in addition (0.24 ± 0.01 and 0.33 ± 0.05/μm, respectively). Similarly, desmosome size was significantly reduced after incubation with PV-IgG alone as compared to control samples (Figure 2C). However, in samples which received UO126 together with PV-IgG, both desmosome number and size were reduced but reduction was not significant compared to controls. We further investigated other ultrastructural hallmarks including altered keratin filament association and split desmosomes, and assessed whether these conditions can be rescued by inhibiting PKC or ERK signaling. In the control samples, very few desmosomes with poor keratin filament association were observed and these may be related to nascent desmosomes (33). Keratin filament insertion was severely altered in all samples incubated with PV-IgG in presence or absence of PKC and ERK signaling inhibitors (Figure 2D). Next, we quantified the number and length of split desmosomes in the vicinity of blisters in all samples and for each condition (Figure 3). There was no split desmosomes in control samples but present in all samples which received PV-IgG with or without either of the inhibitors (Figures 3A,C). Moreover, split desmosomes were found to be significantly reduced in size under all conditions compared to normal desmosomes in controls (Figure 3B).

**Figure 4:**
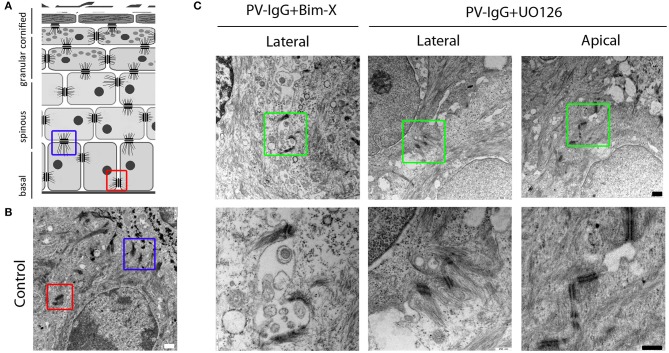
Desmosome alterations at the lateral and apical interfaces of basal keratinocytes. **(A,B)** Schematic diagram and control electron-micrograph (scale bar 500nm) showing the apical (blue box) and lateral (red box) desmosomes. **(C)** Electron-micrographs of samples treated with PV-IgG with either Bim-X or UO126. Scale bars for upper and lower panels, 500 and 250 nm, respectively.

Finally, since we observed that large blisters were absent in samples treated with PV-IgG together with UO126 but not with Bim-X and rather small clefts were present between basal cells (Figure 1A), we compared lateral and apical desmosomes of basal keratinocytes under these conditions (Figures 4A,B). Lateral desmosomes were similar in samples incubated with PV-IgG together with Bim-X or with UO126 and interdesmosomal widening was observed as well. In contrast, the apical desmosomes between basal and suprabasal cells, which were absent in conditions using PV-IgG alone or with Bim-X due to blister formation, were largely preserved in samples incubated with both PV-IgG and UO126 (Figure 4C).

## Discussion

In the current investigation, we tested the role of PKC and ERK signaling in blister formation and ultrastructural alterations of desmosomes induced by PV-IgG in human *ex vivo* skin model.

### Inhibition of ERK but Not of PKC Is Effective to Block Blister Formation in *ex vivo*

From the histological analysis, inhibition of PKC activity was not protective against blister formation. In contrast, inhibition of MEK, which is the up-stream kinase of ERK1/2, using UO126 was sufficient to ameliorate epidermal blistering in the skin samples.

Using a similar model, blister formation in human skin has been sufficiently blocked by inhibition of p38MAPK signaling (18) whereas in mucosa explant culture inhibition of p38MAPK was not protective against PV-IgG-induced blistering (19). Previous experiments have demonstrated that inhibition of PKC and ERK pathways sufficiently abrogated cell dissociation in keratinocyte cell cultures (17, 20, 25) and deactivation of PKC signaling in neonatal pemphigus mouse model effectively blunted blister formation (30, 31). Similarly, specific inhibition of Src or its upstream targets have been shown to prevent keratinocyte cell dissociation in cultures (17, 34, 35) as well as blistering in mouse models (34, 36, 37). However, Src inhibition in human skin explant culture was not effective to avert epidermal blistering as well (34). Therefore, our data suggest that the role of different signaling pathways, which are involved in regulation of desmosomal adhesion in cultured keratinocytes, is not identical in neonatal mouse skin and adult human epidermis. Thus, the study underscores the importance of evaluating a given signaling pathway in intact human skin before any recommendation for therapeutic application is considered.

### Erk Prevents Loss of Desmosomes in the Suprabasal Cleavage Plane of the Epidermis

Our ultrastructural analysis shows that specific inhibition of PKC or ERK signaling did not abrogate the ultrastructural hallmarks characteristic of pemphigus including shortening and splitting up of desmosomes as well as alterations in keratin association with desmosomal plaques. Inhibition of ERK signaling, however, significantly prevented decrease in desmosome number and blocked blister formation. This is in agreement with the finding that acantholysis in human epidermis correlated with reduction in desmosome number rather than with desmosome size (18). Similarly, ultrastructural evaluation of pemphigus patients' lesional skin asserted that blister formation is paralleled by loss of desmosomes (32). Because inhibition of p38MAPK was sufficient to block blistering and rescue desmosome ultrastructure in human skin explants, we assumed a crucial role of p38MAPK in desmosome and cytoskeletal regulation (18). However, Src inhibition didn't avert decrease in desmosome number and size in human skin culture (34) which is similar to our present observations that PKC inhibition was not effective to prevent the ultrastructural damage caused by PV-IgG injection.

*In vivo* experiments in neonatal mouse have shown that desmosomes are split before they are eventually internalized (38). We observed split desmosomes in keratinocytes at blister interfaces as well as at sites where the cleavage begins or ends, similar to previous investigations using electron microscopy and SIM (18, 32, 39). Desmosomes are split due to the dys-cohesive effects caused by PV-IgG binding (39) which may be attributed to steric hindrance. In addition, the fact that split desmosomes were significantly reduced in size in our study may suggest that desmosomal components were depleted in this process, which at least in part may be caused by signaling mechanisms. However, formation of split desmosomes in the vicinity of epidermal blisters was not altered by inhibition of PKC or ERK signaling, although inhibition of ERK signaling significantly reduced fragmentation of Dsg1 staining. This observation indicates that other desmosomal components including Dsg3 may also be important to preserve desmosome integrity. These data suggest that Dsg1 depletion is regulated by ERK whereas Dsg3 depletion is caused by mechanisms independent of both PKC and ERK. The latter is different to a previous study in which a PV-IgG fraction from another patient caused Dsg3 depletion in PKC-dependent manner (30).

Interestingly, in samples treated with UO126 followed by PV-IgG injection, we observed that apical desmosomes between basal and suprabasal cells were preserved, which correlated with the absence of large epidermal blisters under this condition, whereas lateral desmosomes of basal cells were altered similar to conditions in which PKC was inhibited parallel to autoantibody incubation. Differences in the distribution and composition of desmosomal structures between the lateral and apical surfaces of basal keratinocytes was reported in an *in vivo* study in which nascent desmosomes were seen in lateral surfaces but not at apical cleavage sites during acantholysis (33). Moreover, it is known that epidermal cells exhibit a layer-dependent distribution of desmosomal proteins and show a layer-specific signal modulation. For example, EGFR is concentrated at the basal and suprabasal layers where actively proliferating and differentiating cells are present (22). In addition, Dsg1 has a unique expression pattern in that in basal cells it is confined largely to the apical surface and thus the interface to suprabasal cells (22), which means that Dsg1 is largely absent at the lateral interfaces of basal cells. Because we observed that ERK activation by pemphigus autoantibodies correlated with presence of autoantibodies targeting Dsg1 but not with expression of Dsg3 in keratinocytes (17, 21), we speculate that inhibition of ERK signaling may have been effective to ameliorate skin blistering at least in part via stabilization of apical desmosomes of basal keratinocytes. This is intriguing since this is the epidermal cleavage plane in PV. In line with this, we observed that after inhibition of ERK signaling, localization of Dsg1 in the basal epidermis was partially rescued compared to samples treated with PV-IgG alone. In contrast, PKC inhibition did not prevent blister formation and was not effective to reduce Dsg1 depletion in basal layers of the epidermis.

In summary, *ex vivo* models are helpful to evaluate the relative contribution of signaling pathways to blister formation, especially because cell culture models and mice often show complete inhibition of blistering when a single pathway is modulated (17, 30, 31, 37, 40). Differences to the animal studies may be explained by differences in desmosome regulation between neonatal mice and adult human skin. Therefore, electron microscopy studies are important to delineate the role of signaling pathways in the regulation of desmosome ultrastructure.

## Data Availability Statement

The raw data supporting the conclusions of this manuscript will be made available by the authors, without undue reservation, to any qualified researcher.

## Ethics Statement

The studies involving human participants were reviewed and approved by Ethics Committee of the University of Würzburg Medical Faculty. The patients/participants provided their written informed consent to participate in this study.

## Author Contributions

DE performed the experiments and analyzed the data. DK performed and interpreted immunofluorescence data. DE and JW designed the study, interpreted the data, and wrote the manuscript.

### Conflict of Interest

The authors declare that the research was conducted in the absence of any commercial or financial relationships that could be construed as a potential conflict of interest.
